# Functional characterization of the three *Drosophila* retinal degeneration C (RDGC) protein phosphatase isoforms

**DOI:** 10.1371/journal.pone.0204933

**Published:** 2018-09-28

**Authors:** Olaf Voolstra, Lisa Strauch, Matthias Mayer, Armin Huber

**Affiliations:** Department of Biochemistry, Institute of Physiology, University of Hohenheim, Stuttgart, Germany; University of Florida, UNITED STATES

## Abstract

*Drosophila* retinal degeneration C (RDGC) is the founding member of the PPEF family of protein phosphatases. RDGC mediates dephosphorylation of the visual pigment rhodopsin and the TRP ion channel. From the *rdgC* locus, three protein isoforms, termed RDGC-S, -M, and -L, with different N-termini are generated. Due to fatty acylation, RDGC-M and -L are attached to the plasma membrane while RDGC-S is soluble. To assign physiological roles to these RDGC isoforms, we constructed flies that express various combinations of RDGC protein isoforms. Expression of the RDGC-L isoform alone did not fully prevent rhodopsin hyperphosphorylation and resulted in impaired photoreceptor physiology and in decelerated TRP dephosphorylation at Ser936. However, expression of RDGC-L alone as well as RDGC-S/M was sufficient to prevent degeneration of photoreceptor cells which is a hallmark of the *rdgC* null mutant. Membrane-attached RDGC-M displayed higher activity of TRP dephosphorylation than the soluble isoform RDGC-S. Taken together, *in vivo* activities of RDGC splice variants are controlled by their N-termini.

## Introduction

*Drosophila* retinal degeneration C (RDGC) is the founding member of the protein phosphatases with EF hands (PPEF) family. In *rdgC* null mutant flies, rhodopsin becomes light-dependently hyperphosphorylated [1]. This hyperphosphorylation causes light-dependent retinal degeneration and premature entrance into the prolonged depolarizing afterpotential (PDA) [1–3]. A PDA manifests in the persistence of photoreceptor depolarization after cessation of the light stimulus and is caused by an excess of activated rhodopsin (metarhodopsin) molecules over available arrestin molecules. Light-dependent rhodopsin hyperphosphorylation in *rdgC* mutant flies results in a stable interaction between rhodopsin and arrestin 2 molecules. These rhodopsin/arrestin complexes are internalized from the rhabdomeric membranes into the cell body and trigger apoptosis leading to photoreceptor degeneration [4–6]. The stable interaction between arrestin 2 and rhodopsin probably also underlies the premature entrance into the PDA since it reduces the number of available arrestin molecules. RDGC is activated by Ca^2+^ through interaction with calmodulin [7]. RDGC contains an IQ-motif that binds to Ca^2+^/calmodulin and a mutation in this motif, *rdgC*^*I12E*^ (but not *rdgC*^*I12A*^), abolished calmodulin binding and resulted in retinal degeneration [7]. Besides dephosphorylation of rhodopsin, we recently showed that RDGC is also involved in the dephosphorylation of the TRP cation channel at Ser936 [8]. Interestingly, Ser936 is the only known TRP phosphorylation site that is phosphorylated in the dark and becomes dephosphorylated in the light [8–10], consistent with Ca^2+^-dependent activation of RDGC. As revealed by exchange of Ser936-TRP to Ala, preventing phosphorylation, or Asp, mimicking phosphorylation, dephosphorylation of TRP at Ser936 enables photoreceptor cells to process oscillating light stimuli of higher frequencies. Thus, the phosphorylation state of TRP at Ser936 plays a role in the temporal aspect of light adaptation [8].

By comparison, vertebrate PPEF isoforms have mainly been localized to sensory structures such as the inner ear, dorsal root ganglia, embryonic brainstem nuclei, photoreceptors and pinealocytes [11,12] but the role of PPEFs in these neuronal tissues is not known.

The *Drosophila rdgC* gene encodes three protein isoforms, RDGC-S, -M, and -L, that harbor different N-termini. We recently reported that the RDGC-M and RDGC-L protein isoforms are tethered to the plasma membrane due to fatty acylationwhile RDGC-S does not become fatty acylated and is soluble [13]. In this work, we investigated physiological consequences of the different solubilities and subcellular localizations of the RDGC protein isoforms. To do so, we conducted a CRISPR/Cas9 approach to ablate RDGC-S (and RDGC-M) and we used an existing MiMIC fly in which RDGC-L is absent. Expression of RDGC-L alone was not sufficient to fully rescue rhodopsin hyperphosphorylation and resulted in slow pS936-TRP dephosphorylation. Elevated expression of RDGC-M on the other hand, resulted in accelerated pS936-TRP dephosphorylation providing evidence that the N-termini determine the *in vivo* activity of the RDGC protein isoforms.

## Materials and methods

### Fly stocks and illumination conditions

The following *Drosophila melanogaster* strains and mutants were used: *w* Oregon R (here referred to as wild type), *rdgC*^*306*^ [2,3], *yw*;;*ninaE*^*17*^ [14], *yw;*P[*rh1*-*rh1*^*CT S>A*^];*ninaE*^*17*^ [5], *w;P[rh1-rh1*^*Δ356*^];*ninaE*^*17*^ [1], *w*;*arr2*^*3*^,*st* [15], *yw;+;Mi{MIC}rdgC*^*[Mi06989]*^ (Bloomington #42457, here referred to as *rdgC*^*ΔL*^) [16], *y*,M{*vas*-*Cas9*}ZH-2A,*w*/FM7c (Bloomington #51323) [17]. The fly strains *yw*;;*rdgC*^*ΔSM*^ and *yw*;;*rdgC*^*ΔS*^ were generated in this study (see below). All flies were white-eyed.

Flies were illuminated with a 30 watt fluorescent lamp, 2000 lux, unless noted otherwise. To analyze hyperphosphorylation of RH1, flies were kept in the dark over night and then illuminated for 1 h. To investigate retinal degeneration, flies were subjected to a 12 h light/12 h dark cycle at 25 °C and were illuminated with a 30 watt fluorescent lamp, 1200 lux. To investigate long-term dephosphorylation of TRP at S936, flies were illuminated or dark adapted over night and were then subjected to the opposite light condition for 1 h. To investigate pS936-TRP dephosphorylation kinetics, flies were dark adapted over night and were subsequently illuminated with a white 350 mA light-emitting diode, 35,000 lux, for time spans ranging from 10 to 300 sec.

### Generation of flies lacking the small RDGC protein variants

For ablation of RDGC-S (and RDGC-M) protein variants, the genomic region of the first coding exon of transcript *rdgC-RB*(see S1 Fig) was targeted in a CRISPR-Cas9 approach. Two oligonucleotides, forward, 5’-GTCGTGCATCAAAATGGATGAGAA-3‘ and reverse, 5’-AAACTTCTCATCCATTTTGATGCA-3’ were hybridized and cloned into a BbsI-digested pCFD3-dU6:3gRNA vector [18]. The plasmid construct was injected (1 μg/μl) into flies expressing Cas9 under control of the *vasa* promotor (Bloomington #51323) [17]. Injected flies were crossed to *rdgC*^*306*^ flies. Offspring was crossed to the *rdgC*^*306*^ mutant and was analyzed by Western blotting for deviations in the RDGC protein band pattern. The resulting flies are referred to as *rdgC*^*ΔS*^ and *rdgC*^*ΔSM*^, respectively.

### Western blot analyses

For Western blot analyses, fly heads were homogenized in 1× SDS extraction buffer (4% SDS, 1 mM EDTA, 75 mM Tris/HCl, pH 6.8), and extraction was carried out for 10 min at room temperature. In case of λ-phosphatase (New England Biolabs) treatment, fly heads were homogenized in extraction buffer (50 mM Tris-HCl, pH 8.0, 150 mM NaCl, 1% Triton X-100). λ-phosphatase-negative controls were homogenized in extraction buffer supplemented with phosphatase inhibitors (10 mM sodium fluoride, 0.1 mM orthovanadate). Extraction was carried out for 30 min at 4 °C. The reaction was done according to the manufacturer’s instructions. The extracts were spun at 16,000 × g at 22 °C for 10 min. Supernatants were subjected to SDS-PAGE using 8 or 12% polyacrylamide gels. Blotting, blocking, incubation with antibodies and signal detection was done as described previously [13]. Quantification of Western blot signals was done with Image Lab 5.2 (Bio-Rad) by integration of the pixel intensities of each band. Background was subtracted using a “rolling disk” size of 10 mm.

### Immunocytochemistry of fly eyes

Immunocytochemistry of fly eyes was carried out as described before [19], with modifications. After blocking, sections were incubated with Alexa Fluor 546-coupled Phalloidin (Invitrogen, 1:600) in 1% BSA and 0.3% Triton X-100 in PBS for 2 h. Images were acquired using a Plan-Neofluar x40/1.3 oil objective.

### Assessment of the deep pseudopupil

The deep pseudopupil was recorded with a DFC420 C camera (Leica) mounted on a MZ 16 F stereo microscope (Leica). A KL 1500 LCD cold light source (Schott) with a ring light adaptor was used for illumination.

### Electroretinogram recordings

Electroretinograms were recorded with an EXT 10-2F amplifier (npi electronic) with a 700 Hz low pass filter applied. Blue and orange light emitting diodes (LEDs, Roithner M3L1-HB-30 and M3L1-HY-30) served as light sources. Light intensities were adjusted using neutral density to 19 μW/cm^2^ for the orange light and to 2 μW/cm^2^ for the blue light. The LEDs were driven by a PLED-02M driver (npi electronic). A USB-6211 card (National Instruments) was used for analog to digital conversion. The card was connected to a standard personal computer running the WinWCP 4.7.6 software (Strathclyde University). Electrodes were pulled from capillaries with an outer diameter of 1.5 mm and an inner diameter of 0.75 mm, with filament (Sutter BF150-75-10) using a Narishige PC-10 puller. Glass electrodes were filled with Davenport solution (100 mM NaCl, 2 mM KCl, 1 mM CaCl_2_, 1.8 mM NaHCO_3_, pH 7.2). Silver wires (250 μm diameter, GoodFellow) were chlorided with an ACl-01 automatic chlorider (npi electronic) and placed into the Davenport-filled glass capillaries. The electrodes had a resistance of ~30 mΩ (when immersed in a 0.9% NaCl solution). The recording electrode was inserted into the eye just below the cornea. The reference electrode was inserted into the head capsule.

### Antibodies

The polyclonal α-RH1, α-RDGC, and α-pS936-TRP antibodies were described before [9,13,20]. Monoclonal α-RH1 (4C5), α-TRP (Mab83F6), and α-beta-Tubulin (E7), antibodies were obtained from the Developmental Studies Hybridoma Bank. Secondary antibodies for detection of the ECL signal were as described [13].

## Results

### Generation of flies expressing different combinations of RDGC isoforms

**Fig 1:**
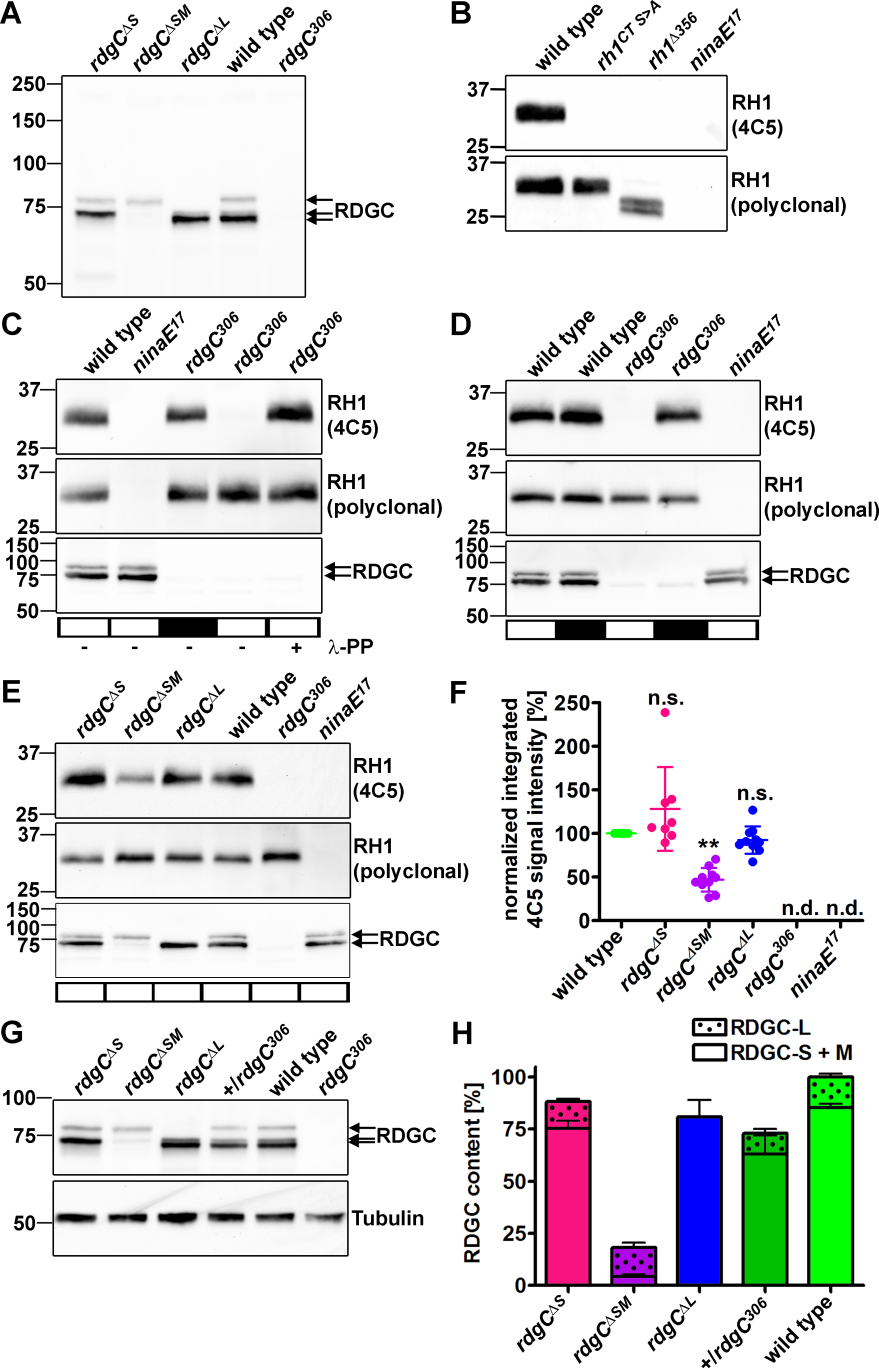
Flies that lack both RDGC-S and RDGC-M isoforms exhibit RH1 hyperphosphorylation. A, Heads of flies lacking RDGC-S (*rdgC*^*ΔS*^), RDGC-S and RDGC-M (*rdgC*^*ΔSM*^), and RDGC-L (*rdgC*^*ΔL*^) as well as from control flies were subjected to Western blot analyses using an α-RDGC antibody. B, In order to confirm the epitope of the monoclonal 4C5 α-RH1 antibody, wild type, *rh1*^*CT S>A*^, *rh1*^*Δ356*^, and *ninaE*^*17*^ fly heads were subjected to Western blot analyses. The *rh1*^*CT S>A*^ fly expresses RH1 in which the putative C-terminal phosphorylation sites were exchanged by Ala and the *rh1*^*Δ356*^ fly expresses a C-terminally truncated RH1. Western blots were probed with the 4C5 α-RH1 antibody (upper panel) and a polyclonal α-RH1 antibody (lower panel). C, Dark-reared flies of the indicated genotypes were illuminated for 1 h (white bars) or kept in the dark (black bar) and head extracts were incubated with (+) or without (-) ʎ-phosphatase (ʎ-PP). Head extracts were then subjected to Western blot analyses using monoclonal (4C5) and polyclonal α-RH1 antibodies as well as an α-RDGC antibody. D, Dark-reared flies of the indicated genotypes were illuminated for 1 h (white bars) or kept in the dark (black bars) and head extracts were subjected to Western blot analyses using monoclonal (4C5) and polyclonal α-RH1 antibodies as well as an α-RDGC antibody. E, *rdgC*^*ΔS*^, *rdgC*^*ΔSM*^, *rdgC*^*ΔL*^, and control flies were reared in the dark and illuminated for one hour before fly heads were subjected to Western blot analysis. A representative blot (E) and the quantification of signals obtained with the 4C5 antibody (F) are shown. The normalized integrated 4C5 signal intensity was calculated by dividing the integrated pixel intensities of the bands obtained with the 4C5 antibody by those of corresponding bands obtained with the polyclonal α-RH1 antibody. Error bars in (F) show SD. The significance was calculated using ANOVA with Dunnett’s multiple comparison test. n.s., not significant; n.d., not detected; ** p ≤ 0.01. G, Eyes of the indicated flies were subjected to Western blot analyses using α-RDGC and α-tubulin antibodies. H, Signals from RDGC-S and -M protein variants or the RDGC-L protein isoforms were quantified and normalized by tubulin signals. N = 6–9, error bars show SEM.

To assign physiological functions to the different RDGC protein isoforms, we generated fly strains that express different combinations of RDGC-S, RDGC-M, and RDGC-L. A fly lacking the RDGC-L variant was obtained from the Bloomington stock center. This fly harbored a minos mediated integration cassette (MiMIC) in the first intron of the *rdgC* locus (*rdgC*^*[Mi06989]*^) [16]. The integration cassette is composed of a splice acceptor site followed by stop codons for all three reading frames, the coding sequence of enhanced green fluorescent protein, and a polyadenylation signal sequence [16]. We reasoned that this insertion should result in selective ablation of the RDGC-L protein variant. Indeed, the *rdgC*^*[Mi06989]*^ fly solely expressed the RDGC-S and RDGC-M protein isoforms but not RDGC-L (Fig 1A), and will hereafter be referred to as *rdgC*^*ΔL*^. To obtain a fly expressing the RDGC-L protein variant but lacking RDGC-S (and possibly RDGC-M), we targeted the first exon of the *rdgC-S* variant in a CRISPR/Cas9 approach. In one of the obtained mutant strains, RDGC-S was absent and RDGC-M was drastically reduced (Fig 1A). Sequencing of genomic DNA revealed a five base pair deletion resulting in a frameshift (S1 Fig). This fly will hereafter be referred to as *rdgC*^*ΔSM*^. The five base pair deletion in the *rdgC*^*ΔSM*^ fly allows generation of *rdgC-S* mRNA but prevents synthesis of the RDGC-S protein due to the introduced frame shift. We obtained another fly strain lacking RDGC-S and expressing elevated amounts of RDGC-M (Fig 1A). This fly will hereafter be referred to as *rdgC*^*ΔS*^. Sequence analysis of genomic DNA revealed a 21 base pair deletion destroying both the start codon of *rdgC*-S and the splice donor site of the first exon (S1 Fig). We speculate that the respective pre-mRNA is exclusively spliced to *rdgC-M* mRNA resulting in elevated RDGC-M protein levels (see Fig 1A).

### The 4C5 α-RH1 antibody can be used to monitor rhodopsin hyperphosphorylation

Monoclonal α-rhodopsin antibodies raised against the C-terminus of bovine rhodopsin have been reported to poorly bind strongly phosphorylated rhodopsin [21]. Using a fly that expresses a C-terminally truncated RH1 lacking the last 17 amino acids (*rh1*^*Δ356*^, [1]), Alloway and colleagues showed that the epitope of the monoclonal 4C5 antibody overlaps with this region [6]. To confirm this finding, we probed head extracts from these flies and from flies expressing RH1 in which the putative phosphorylation sites were mutated (*rh1*^*CT S>A*^, [5]) in Western blot analyses (Fig 1B). We found that the 4C5 antibody did not detect either of the modified RH1 proteins suggesting that the epitope of this antibody indeed overlaps with the region that is phosphorylated. Taken together with the observations made by Adamus and colleagues on bovine rhodopsin [21], the 4C5 antibody has the potential to indicate hyperphosphorylation of RH1 in *Drosophila* by reduced binding. It is known that RH1 becomes hyperphosphorylated in *rdgC* null mutants upon illumination [1]. The 4C5 antibody failed to detect RH1 from illuminated *rdgC*^*306*^ flies while it readily detected RH1 from dark-reared *rdgC*^*306*^ flies (Fig 1C). In contrast, the 4C5 antibody detected RH1 in light-adapted wild type flies. To provide direct evidence that failure of the 4C5 antibody to bind to RH1 from illuminated *rdgC*^*306*^ is caused by hyperphosphorylation, we incubated head extracts from illuminated *rdgC*^*306*^ flies with ʎ-phosphatase to remove phosphate groups. Indeed, upon incubation with ʎ-phosphatase, the 4C5 antibody detected rhodopsin from illuminated *rdgC*^*306*^ fly head extracts. Collectively, these data suggest that the monoclonal 4C5 α-RH1 antibody indicates hyperphosphorylation of RH1 through impaired binding. To investigate possible differences with regard to 4C5 antibody binding to RH1, we illuminated dark-reared flies for 1 h (Fig 1D, white bars) or kept them in the dark (Fig 1D, black bars), and subjected fly heads to Western blot analyses. Signal intensities obtained with the 4C5 antibody were similar in head extracts from dark-reared and illuminated wild type flies. In contrast, illumination resulted in a strong reduction of 4C5 signal intensity in *rdgC*^*306*^ flies. Thus, the majority of epitopes detected by the 4C5 antibody is destroyed when *rdgC*^*306*^ flies are illuminated, presumably by phosphorylation, while the majority of epitopes is left intact upon illumination of wild type flies. To further characterize the binding of the 4C5 α-RH1 antibody to RH1, we mixed head extracts from illuminated wild type and *rdgC*^*306*^ flies to vary the content of hyperphosphorylated RH1 but ensure equal overall RH1 amounts. The protein extracts were subjected to Western blot analysis (S2A Fig). Increasing amounts of wild type head extracts led to an exponential increase of the signal intensity. This observation might be explained by the notion that hyperphosphorylated RH1 molecules that are not bound by the 4C5 antibody exert an additional effect in masking epitopes from normally phosphorylated RH1 molecules. Thus, by increasing the proportion of normally phosphorylated RH1 (from wild type head extracts) and decreasing the proportion of hyperphosphorylated RH1 (from *rdgC*^*306*^ head extracts), a greater number of epitopes is present, concomitant with less epitopes being masked by disrupted hyperphosphorylated epitopes. Using sample preparation conditions that result in the formation of rhodopsin multimeres, we tested whether or not rhodopsin dimers obtained from light-adapted *rdgC*^*306*^ flies are detected by the 4C5 antibody (S2C Fig). Our finding showing that this is not the case excludes the possibility that the lack of 4C5 signal in rhodopsin monomers results from enhanced formation of rhodopsin multimeres in *rdgC*^*306*^ flies.

### RDGC-L alone does not fully rescue RH1 dephosphorylation

To test *rdgC*^*ΔS*^, *rdgC*^*ΔSM*^, and *rdgC*^*ΔL*^ flies for possible hyperphosphorylation of RH1, we illuminated them for one hour and subjected fly heads to Western blot analysis using the 4C5 α-RH1 antibody (Fig 1E). Signal intensities obtained with the 4C5 antibody were normalized to signal intensities obtained with a polyclonal α-RH1 antibody. While normalized 4C5 signal intensities from *rdgC*^*ΔS*^, *rdgC*^*ΔL*^, and wild type flies were indistinguishable, signal intensities from illuminated *rdgC*^*ΔSM*^ flies were reduced to 46.9% +/- 13.6% as compared to wild type flies (Fig 1F). To correlate these observations to RDGC protein levels, RDGC signals were normalized to Tubulin signals (Fig 1G and 1H). RDGC-L and RDGC-S/M signal intensities could readily be determined separately, but it was not possible to quantify RDGC-S and RDGC-M separately due to their similar electrophoretic mobility. Quantification revealed that total RDGC content was drastically lowered in *rdgC*^*ΔSM*^ flies, as expected (Fig 1H). Thus, RH1 hyperphosphorylation in these flies might be caused by reduced RDGC amounts. *rdgC*^*ΔS*^ and *rdgC*^*ΔL*^ flies lacking RDGC-S and RDGC-L, respectively did not exhibit RH1 hyperphosphorylation. We conclude that all RDGC variants are capable of dephosphorylating RH1 but ablation of RDGC-S in combination with a reduction of RDGC-M led to partial RH1 hyperphosphorylation.

### *rdgC*^*ΔSM*^ flies display an elevated susceptibility to enter the PDA state

**Fig 2:**
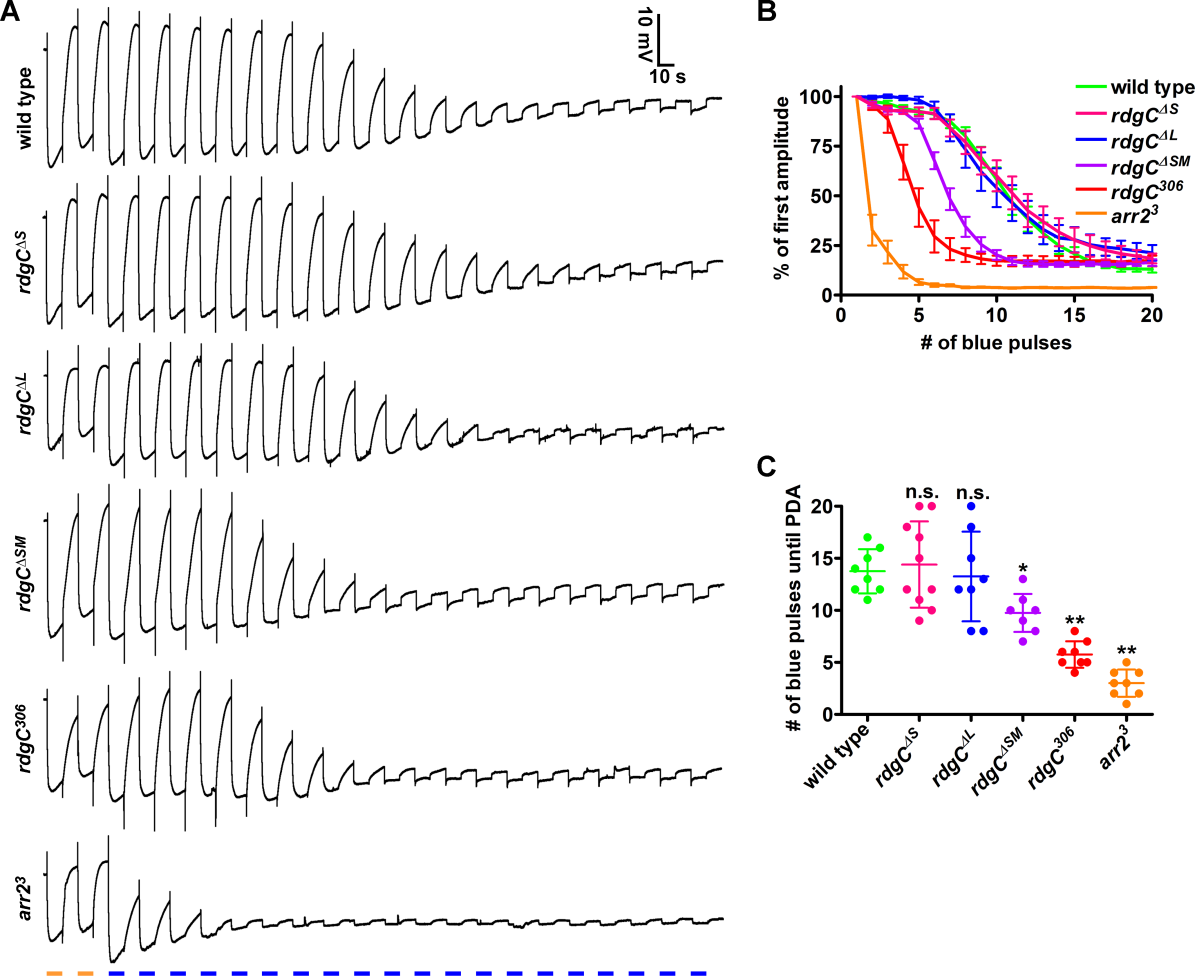
Premature entrance into PDA in *rdgC* mutants showing RH1 hyperphosphorylation. Dark-reared, one day old flies received two orange light pulses and 20 blue light pulses of 10 sec duration with 10 sec pauses between stimuli. A, Representative ERG recordings of flies of the given genotypes. B, Graph showing ERG amplitudes normalized to the amplitude in response to the first blue light pulse. Error bars show SEM. C, Graph showing the number of blue light pulses needed to elicit a PDA. Error bars show SD. The significance was calculated using ANOVA with Dunnett’s multiple comparison test. N = 8–10. n.s., not significant; *, p ≤ 0.05; ** p ≤ 0.01.

It has been shown that a PDA can be elicited in *rdgC* null mutant flies with lower intensities of blue light as compared to wild type flies [1,7]. Another possibility to analyze the susceptibility to enter the PDA state is to use recurrent low intensity blue light pulses [22]. We subjected dark-reared wild type (N = 8), *rdgC*^*ΔS*^ (N = 10), *rdgC*^*ΔL*^ (N = 8), *rdgC*^*ΔSM*^ (N = 8), *rdgC*^*306*^ (N = 8), and *arr2*^*3*^ (N = 8) flies to two orange light pulses (~20 μW/cm^2^) followed by 20 low intensity blue light pulses (~2 μW/cm^2^) and recorded electroretinograms (ERGs) (Fig 2A). *arr2*^*3*^ flies served as a control because *arr2* mutant flies have been already shown to enter the PDA state prematurely under comparable experimental conditions [22]. To quantify the decrease of recovery with an increasing number of blue light pulses, we measured the amplitudes of the ERG traces in response to each blue light stimulus (Fig 2B). We also counted the number of blue light pulses that was needed to elicit a PDA (Fig 2C). We considered the last ERG response showing an on-transient as the starting point of the PDA. In this experimental setup, wild type flies entered the PDA state after 13.75 ± 2.12 blue light pulses. As expected, *arr2*^*3*^ mutant flies exhibited a much stronger decrease of recovery than wild type flies (Fig 2B) and entered the PDA state already after 3 ± 1.3 blue light pulses (Fig 2C). *rdgC*^*306*^ mutants also showed a much stronger decrease of deactivation than wild type flies (Fig 2B) and entered the PDA state after 5.75 ± 1.28 blue light pulses (Fig 2C). We did not observe differences between wild type and *rdgC*^*ΔL*^ or *rdgC*^*ΔS*^ flies. Interestingly, the *rdgC*^*ΔSM*^ fly that exhibited partial RH1 hyperphosphorylation, also displayed a stronger amplitude degradation than the wild type (Fig 2B). Consistently, the number of blue light pulses needed to elicit a PDA (9.75 ± 1.83) was also reduced in this fly (Fig 2C). Collectively, these data suggest that the partial RH1 hyperphosphorylation of the *rdgC*^*ΔSM*^ fly manifests physiologically in a higher susceptibility to enter the PDA state. As has already been suggested for the elevated PDA susceptibility of the *rdgC*^*306*^ null mutant [1], we propose that this is caused by an enhanced interaction between RH1 and ARR2 reducing the amount of available ARR2 molecules to switch off activated RH1 after cessation of the light stimulus.

### Hyperphosphorylation of RH1 in *rdgC*^*ΔSM*^ flies does not evoke retinal degeneration

**Fig 3:**
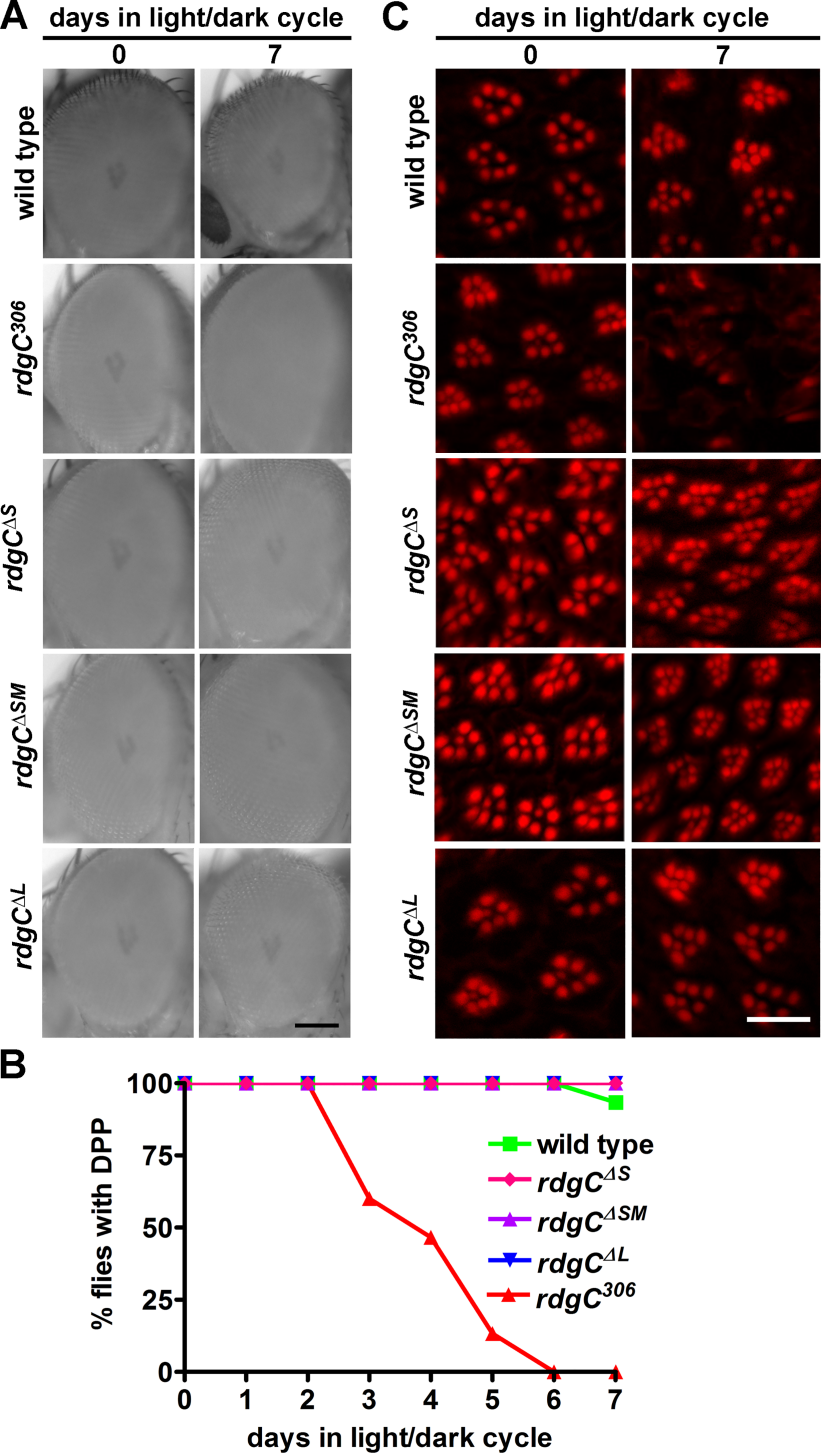
Retinal degeneration in *rdgC* mutants showing RH1 hyperphosphorylation. Retinal degeneration of flies with altered RH1 phosphorylation was assessed by different readouts. A, Representative images of the deep pseudopupils (DPP) of flies with the indicated genotypes after 0 and 7 days in a 12 h light/12 h dark cycle. Scale bar, 100 μm. B, Percentage of flies exhibiting a DPP after 0 to 7 days in the light/dark cycle. For each time point, at least 12 flies were imaged. C, Cross sections through the eyes of *rdgC*^*ΔS*^, *rdgC*^*ΔSM*^, *rdgC*^*ΔL*^, and control flies after 0 or 7 days in the light/dark cycle. AlexaFluor546-coupled phalloidin was used to label the rhabdomeres. Scale bar, 10 μm.

Hyperphosphorylation of rhodopsin results in the generation of stable rhodopsin-arrestin complexes that are internalized, activate apoptosis, and ultimately cause retinal degeneration [5,6]. We therefore asked whether the partial hyperphosphorylation of RH1 in *rdgC*^*ΔSM*^ flies results in retinal degeneration. We monitored retinal degeneration by two different methods (Fig 3). First, we assessed the formation of the deep pseudopupil (DPP). The DPP is a superposition of virtual images of the rhabdomeres of adjacent ommatidia that can easily be viewed with a low power microscope focused at a plane below the photoreceptor cells under orthodromic illumination [23,24] (Fig 3A). As expected, *rdgC* null mutant flies exhibited loss of the DPP after three days in a 12 h light/12 h dark cycle (Fig 3B). After six days, none of the *rdgC*^*306*^ flies exhibited a DPP. In contrast, *rdgC*^*ΔS*^, *rdgC*^*ΔSM*^, and *rdgC*^*ΔL*^ flies preserved their DPP over a period of seven days. We also monitored the rhabdomere structure through phalloidin staining of cryosections (Fig 3C). Phalloidin binds to F-actin and labels the rhabdomeres. We found that rhabdomeres of *rdgC*^*306*^ flies were initially intact but were disrupted after seven days in the light/dark cycle. In contrast, rhabdomeres of *rdgC*^*ΔS*^, *rdgC*^*ΔSM*^, and *rdgC*^*ΔL*^ flies remained intact after seven days in the light/dark cycle. Taken together, the *rdgC*^*ΔS*^, *rdgC*^*ΔSM*^, and *rdgC*^*ΔL*^ flies did not exhibit retinal degeneration within the time window in which *rdgC*^*306*^ null mutant photoreceptors fully degenerated. The finding that photoreceptor cells of *rdgC*^*ΔL*^ did not degenerate is in line with previous findings showing that a genomic rescue construct expressing RDGC-S and -M in an *rdgC* null mutant background rescued retinal degeneration [3,7].

### RDGC-M displays fast kinetics in the dephosphorylation of pS936-TRP

**Fig 4:**
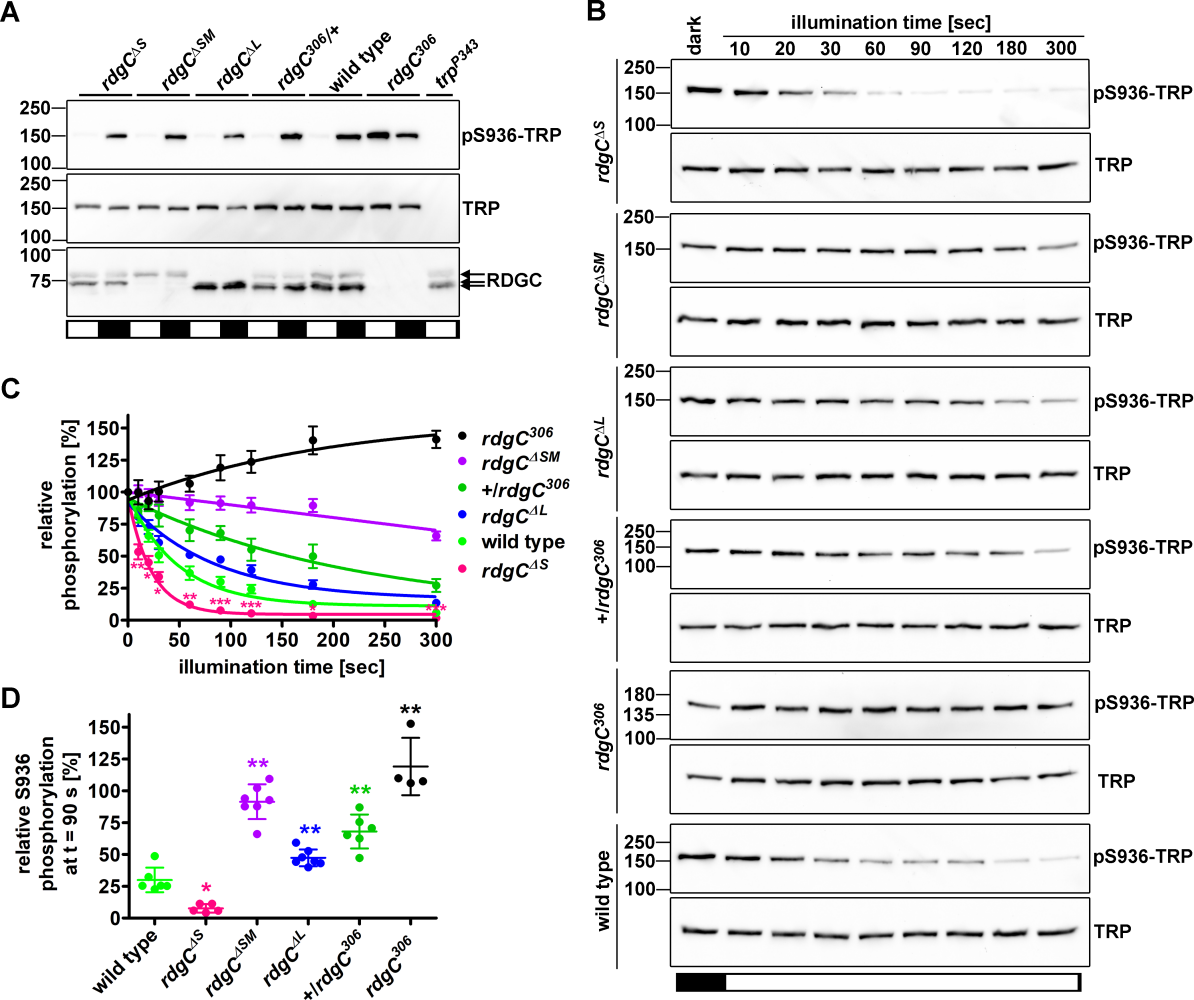
Dephosphorylation of pS936-TRP is delayed in *rdgC*^*ΔSM*^ and *rdgC*^*ΔL*^ flies but is accelerated in *rdgC*^*ΔS*^ flies. A, Flies were light or dark adapted for 1 h before heads were subjected to Western blot analyses using α-pS936, α-TRP, and α-RDGC antibodies. B, Flies were dark adapted over night and then illuminated for various durations before heads were subjected to Western blot analyses. C, Signals obtained with the α-pS936-TRP antibody were normalized by those obtained with the α-TRP antibody and plotted as % of the values obtained at t = 0 s. N = 4–7, error bars show SEM. Asterisks indicate significance levels of differences between wild type and *rdgC*^*ΔS*^ for the respective time points as calculated by Student’s t-test. *, p ≤ 0.05; **, p ≤ 0.01; ***, p ≤ 0.001. D, Scatter plot showing relative S936 phosphorylation after 90 s of illumination as % of t = 0 s. Error bars show SD. The significance was calculated using ANOVA with Dunnett’s multiple comparison test. *, p ≤ 0.05; ** p ≤ 0.01.

Recently, we reported light- and RDGC-dependent dephosphorylation of TRP at Ser936 [8]. To investigate the general capacity of the *rdgC*^*ΔS*^, *rdgC*^*ΔSM*^, and *rdgC*^*ΔL*^ flies to dephosphorylate pS936-TRP, we light and dark adapted flies for one hour and subjected fly heads to Western blot analyses (Fig 4A). Using a phosphospecific antibody that was raised against a phosphorylated synthetic peptide resembling TRP phosphorylated at S936 [8–10], we found that TRP was dephosphorylated in the light and phosphorylated in the dark at S936 in *rdgC*^*ΔS*^, *rdgC*^*ΔSM*^, and *rdgC*^*ΔL*^ fly heads like in the wild type. Thus, RDGC-M and -L (*rdgC*^*ΔS*^), RDGC-L alone (*rdgC*^*ΔSM*^), as well as RDGC-S and -M (*rdgC*^*ΔL*^) are generally capable to mediate TRP dephosphorylation at S936 in the light. To examine a possible impact on the speed of pS936-TRP dephosphorylation when the RDGC-S or RDGC-S/M or RDGC-L isoforms are reduced, we illuminated dark-adapted *rdgC*^*ΔS*^, *rdgC*^*ΔSM*^, and *rdgC*^*ΔL*^ flies for different time spans, and monitored the phosphorylation state of S936-TRP by Western blot analyses (Fig 4B). pS936-TRP signals were normalized to total TRP contents and plotted (Fig 4C). To correlate the kinetics to RDGC contents, we quantified the amounts of RDGC in the different fly mutants using Western blot analyses (see Fig 1H). As a result, dephosphorylation of pS936-TRP was drastically slowed down in the *rdgC*^*ΔSM*^ fly which can probably be explained by a drastically lowered overall RDGC content in this fly. +/*rdgC*^*306*^ flies that harbor only one intact *rdgC* allele, express ~73% overall RDGC protein as compared to the wild type. In these flies, dephosphorylation kinetics of pS936-TRP are also considerably slowed down. The *rdgC*^*ΔL*^ fly that expresses comparable amounts of RDGC-S and RDGC-M as the wild type, but lacks RDGC-L, also displays slower pS936-TRP dephosphorylation kinetics as compared to the wild type. Absence of RDGC in *rdgC*^*306*^ flies resulted in an increase in S936-TRP phosphorylation upon illumination. We speculate that in the light, kinases that phosphorylate S936-TRP are activated. RDGC counteracts the activity of these kinases. Interestingly, the *rdgC*^*ΔS*^ fly shows the fastest pS936-TRP dephosphorylation kinetics although it does not express higher overall RDGC amounts than the wild type (see Fig 1H). However, in this fly, the proportion of RDGC-M is drastically increased compared to the wild type while RDGC-S is absent. This finding indicates that, *in vivo*, RDGC-M has a higher activity of dephosphorylating pS936-TRP than RDGC-S.

## Discussion

Phosphoprotein phosphatases are known to be multifunctional. We recently showed that the *Drosophila* RDGC phosphatase, besides dephosphorylation of rhodopsin, mediates the dephosphorylation of the TRP ion channel at S936 [8]. Light-dependent Ca^2+^ influx into the photoreceptor cells activates RDGC. Ca^2+^/calmodulin binds to an IQ motif and Ca^2+^ possibly binds directly to the EF hands [7]. These motifs are present in all three RDGC protein isoforms. RDGC activity is elevated after activation of the visual signaling cascade via Ca^2+^ influx through TRP channels resulting in enhanced dephosphorylation of rhodopsin and TRP (at the S936 site) [1,8]. While RDGC-L is apparently dispensable for RH1 dephosphorylation, lack of RDGC-S and -M results in RH1 hyperphosphorylation although not to an extent that is observed in *rdgC* null mutant flies. It has to be noted, though, that RDGC-S is by far the most abundant RDGC variant resulting in drastically reduced overall RDGC levels in the *rdgC*^*ΔSM*^ fly. Expression of RDGC-L (in *rdgC*^*ΔSM*^ flies) or RDGC-S and -M (in *rdgC*^*ΔL*^ flies) is sufficient to ensure long-term dephosphorylation of pS936-TRP. However, in both cases, the kinetics of TRP dephosphorylation is delayed. The phosphorylated C-termini of both rhodopsin and TRP are located intracellularly, and are thus potentially accessible by both soluble RDGC-S and membrane-bound RDGC-M/L. Thus, the RDGC-S variant, although soluble, has still the potential to enter the cytosolic portion of the rhabdomeric microvilli. We observed accelerated kinetics of pS936-TRP dephosphorylation in the *rdgC*^*ΔS*^ fly lacking RDGC-S but expressing elevated amounts of RDGC-M. We conclude that, *in vivo*, RDGC-M has a higher activity compared to RDGC-S. This difference could stem from a generally different catalytic activity of RDGC-M and -S or result from the membrane attachment of RDGC-M that potentially brings this RDGC variant into closer proximity to its membrane-bound substrates. Furthermore, possible differences in catalytic activity could result from the different N-terminal sequences of RDGC isoforms per se. Binding of Ca^2+^/calmodulin to the IQ motif has been demonstrated to abolish the interaction between the N-terminus and the catalytic domain of RDGC [7]. This interaction between the N-terminus and the catalytic domain has been proposed to control the catalytic activity. Thus, different N-termini might influence the catalytic activity of RDGC. Taken together, our data suggest that usage of alternative N-termini controls the solubility and subcellular targeting of the three *Drosophila* RDGC phosphatase isoforms and ultimately their *in vivo* activity.

## Supporting information

S1 Fig Genomic sequence analysis of the *rdgC*^*ΔS*^ and *rdgC*^*ΔSM*^ flies. To construct flies lacking the RDGC-S and RDGC-M protein variants while retaining the long variant, we pursued a CRISPR/Cas9 approach. We targeted the first exon of the *rdgC-RB* transcript. The guide RNA sequence is highlighted in yellow, the protospacer adjacent motif is highlighted in green. The double strand break site is marked with an arrow. The genomic DNA was sequenced. *rdgC*^*ΔS*^ flies exhibited a 21 base pair deletion destroying the start codon and the splice donor site. *rdgC*^*ΔSM*^ flies exhibited a five base pair deletion.
(TIF)

S2 Fig Characterization of the 4C5 α-RH1 antibody. A, To investigate the effect of hyperphosphorylated RH1 on 4C5 α-RH1 antibody binding, head extracts from illuminated wild type and *rdgC*^*306*^ flies were mixed and subjected to Western blot analysis. B, C, Flies of the indicated genotypes were illuminated (white bars) or kept in the dark (black bars) and fly heads were subjected to Western blot analyses using the monoclonal α-RH1 antibody (4C5). Head extracts in B were not boiled before loading on SDS gels to minimize formation of rhodopsin multimeres (the same procedure was applied for samples shown in Fig 1) while head extracts in C were boiled for 1 min at 95 °C to provoke formation of rhodopsin dimers. Like RH1 monomers, RH1 dimers were not detected by the 4C5 antibody in illuminated *rdgC*^*306*^ flies.
(TIF)

## Abbreviations

CRISPR: clustered regularly interspaced short palindromic repeats
ERG: electroretinogram
MiMIC: minos mediated integration cassette
PDA: prolonged depolarizing afterpotential
PPEF: protein phosphatases with EF hands
RDGC: retinal degeneration C
RH1: rhodopsin 1
TRP: transient receptor potential
