# Supplementary figures and images for: Functional characterization of the three Drosophila retinal degeneration C (RDGC) protein phosphatase isoforms
Source: PLoS One. 2018 Sep 28;13(9):e0204933. doi: 10.1371/journal.pone.0204933 (PMC6161916; doi:10.1371/journal.pone.0204933)

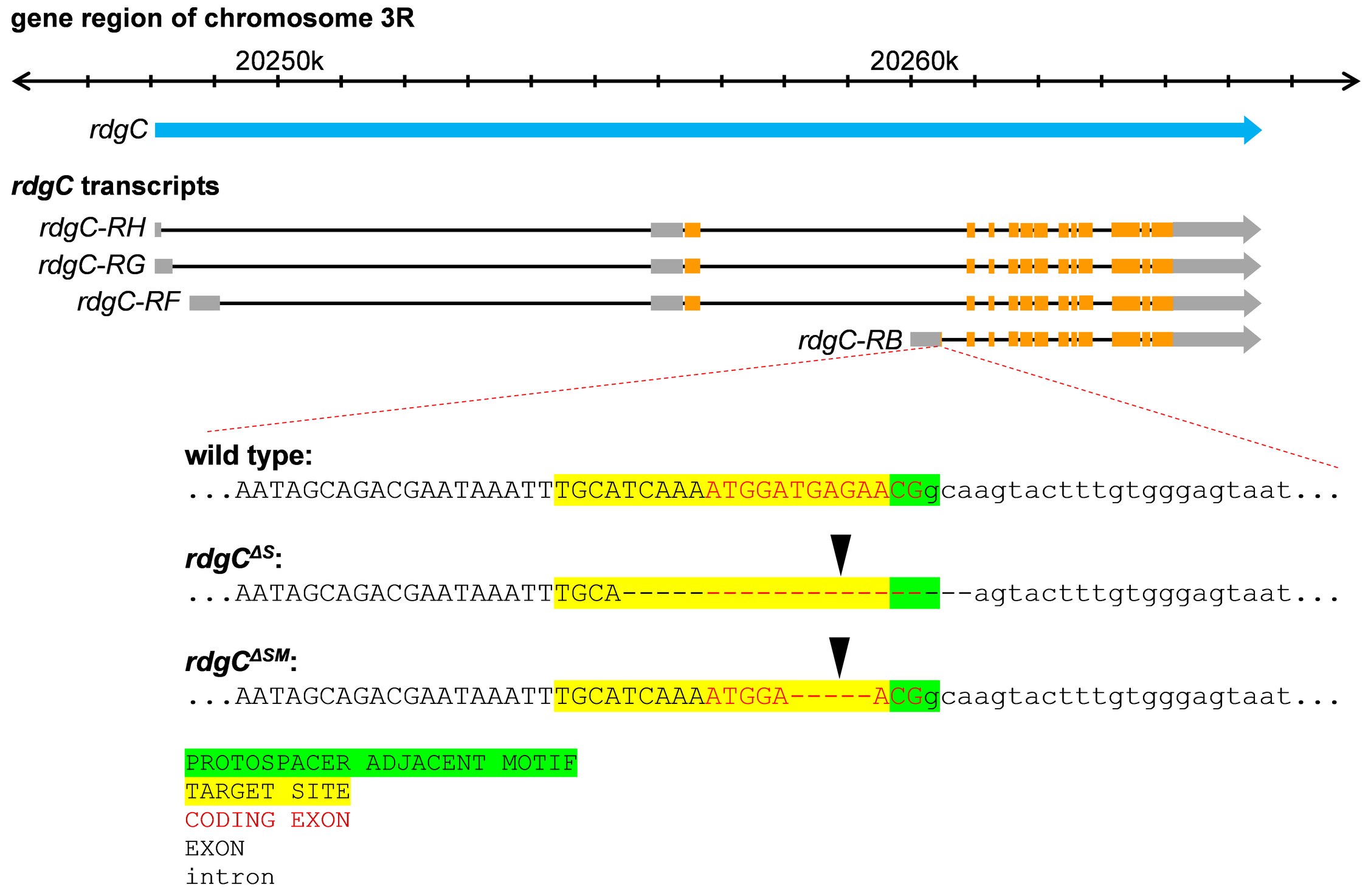

Supplement: S1 Fig — To construct flies lacking the RDGC-S and RDGC-M protein variants while retaining the long variant, we pursued a CRISPR/Cas9 approach. We targeted the first exon of the rdgC-RB transcript. The guide RNA sequence is highlighted in yellow, the protospacer adjacent motif is highlighted in green. The double strand break site is marked with an arrow. The genomic DNA was sequenced. rdgCΔS flies exhibited a 21 base pair deletion destroying the start codon and the splice donor site. rdgCΔSM flies exhibited a five base pair deletion. (TIF) [file pone.0204933.s001.tif]

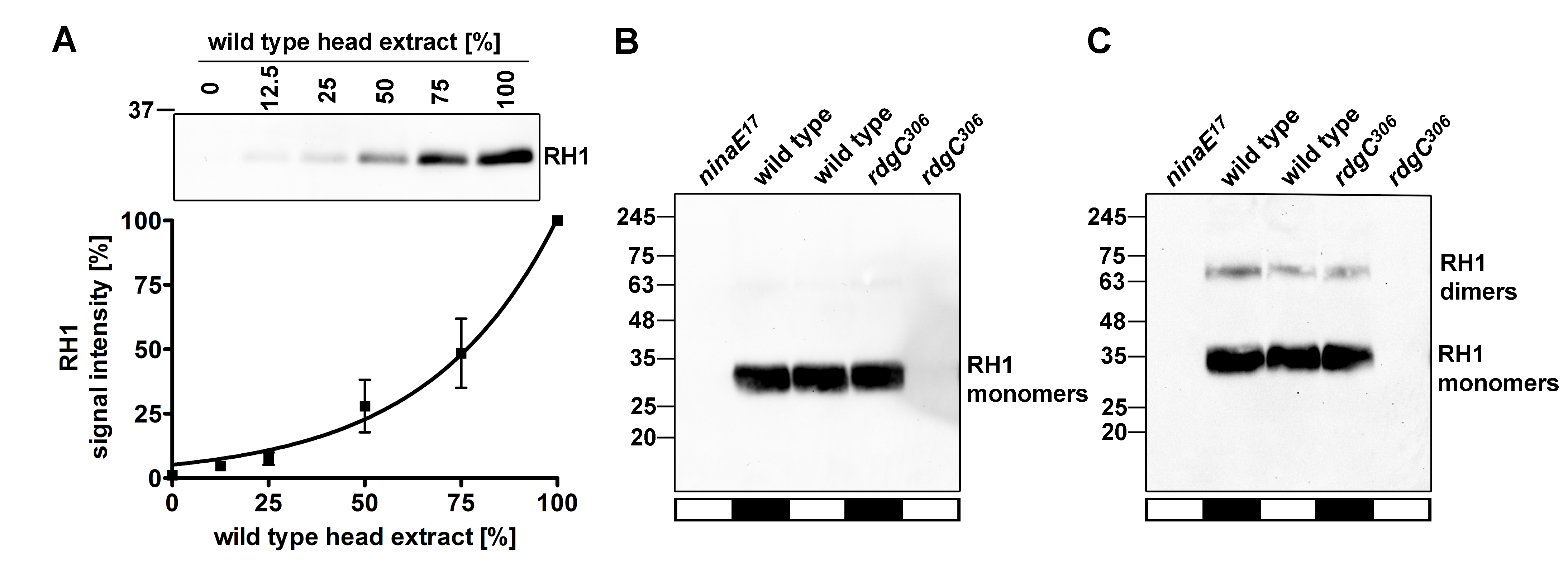

Supplement: S2 Fig — A, To investigate the effect of hyperphosphorylated RH1 on 4C5 α-RH1 antibody binding, head extracts from illuminated wild type and rdgC306 flies were mixed and subjected to Western blot analysis. B, C, Flies of the indicated genotypes were illuminated (white bars) or kept in the dark (black bars) and fly heads were subjected to Western blot analyses using the monoclonal α-RH1 antibody (4C5). Head extracts in B were not boiled before loading on SDS gels to minimize formation of rhodopsin multimeres (the same procedure was applied for samples shown in Fig 1) while head extracts in C were boiled for 1 min at 95 °C to provoke formation of rhodopsin dimers. Like RH1 monomers, RH1 dimers were not detected by the 4C5 antibody in illuminated rdgC306 flies. (TIF) [file pone.0204933.s002.tif]
